# Dr. D. E. S.

**Published:** 1874-09

**Authors:** 


Dr. D. E. S.—"It is impossible to keep up a medical society in our town. A large majority of our physicians are too poor to sacrifice anything to sustain the right when it comes in conflict with their interest. We sometimes talk ethics; but seldom observe its requirements. Every man is for himself, consults with every class of doctors, and charges about what he thinks he can get. . . . We are a free set, with no money, and with little hope of better days, and especially so long as the colored people believe that the Civil Rights bill is intended to purify them and make them superior to the present white race—a people to be fed after the passage of the bill by the ravens, or so long as professors continue to graduate men who no kind of a bill or process could improve. . . . I hope you will excuse my neglect to submit sooner my dues to the Record. I could not do without it, and will be more punctual in the future."

We are sorry to hear even the truth, when the truth bears testimony against the professional irregularities of our brethren, the medical men of the land. We trust the demoralization has not swept away the honor of the profession. We feel that it has not. We are assured, in our esteem and high appreciation of our noble profession, that there are many, very many, true and high-toned professional gentlemen who have not bowed the knee to Baal. We hope they, in concert with all true men of all professions, will seek to elevate the standard of *morality*, and concentrate the good and true into one mighty lens, to fuse and utterly burn corruption from the land.

As to medical colleges, we, with our friends, deplore and lament the sad spectacle. In some sections of our country, they have sprung, fungus-like, into existence, and clamor for patronage.—They resort to every pretext, and hold out inducements for patronage to students in the most indefensible and reprehensible manner, violating every principle of ethics and morals. So far as we are concerned, we can simply look on, and, like Jeramiah, weep over the sad condition of our once-honored profession. We are not attached to any medical school, and we thank God that no responsibility for the evils complained of in every section can be affixed to us. It will be acknowledged by friends and foes that we have been faithful and constant in our efforts against trickery, deception and corruption, as manifested in all the little dishonorable ways and means used for selfish ends.

Having said this much, we take pleasure in saying there are many good institutions worthy of the affection and support of the profession, and we sincerely trust the true men, those looking to the future interest of the brotherhood, will see that they are patronized to the exclusion of barterers and traders in depreciated sheep skins. We give below the names of some of those institutions deserving of professional esteem and support, that have sent us catalogues, but that do not appear in our advertising pages:

Medical College of Georgia, Augusta, Georgia; L. A. Dugas, M.D., Dean.

Savannah Medical College, Savannah, Georgia; W. Duncan, M.D., Dean.

Medical Department of the University of Louisville, Kentucky; J .M. Bodine, M.D., Dean.

Medical Department of the University of Louisiana, New Orleans; T. G. Richardson, M.D., Dean.

Medical Department of the Washington University, Baltimore, Maryland; Chas. W. Chancellor, M. D. Dean.

St. Louis Medical College, St. Louis, Missouri; J. T. Hodgon, M.D., Dean.

Missouri Medical College, St. Louis; John Moore, M.D., Dean.

Department of Medicine and Surgery of the University of Nashville, Tennessee; J. B. Buchanan, M.D., Dean.

Memphis Medical College, Memphis, Tennessee; B. W. Avent, M. D., Dean.

Medical College of South Carolina, Charleston, South Carolina; F. M. Robertson, M.D., Dean.

Jefferson Medical College, Philadelphia, Pennsylvania; J. B. Biddle, M.D., Dean.

Medical College of Virginia, Richmond, Virginia; James B. McCaw, M.D., Dean.

Persons desiring further information, can address the Deans.

